# Absence of Detectable Influenza RNA Transmitted via Aerosol during Various Human Respiratory Activities – Experiments from Singapore and Hong Kong

**DOI:** 10.1371/journal.pone.0107338

**Published:** 2014-09-10

**Authors:** Julian W. Tang, Caroline X. Gao, Benjamin J. Cowling, Gerald C. Koh, Daniel Chu, Cherie Heilbronn, Belinda Lloyd, Jovan Pantelic, Andre D. Nicolle, Christian A. Klettner, J. S. Malik Peiris, Chandra Sekhar, David K. W. Cheong, Kwok Wai Tham, Evelyn S. C. Koay, Wendy Tsui, Alfred Kwong, Kitty Chan, Yuguo Li

**Affiliations:** 1 Alberta Provincial Laboratory for Public Health, University of Alberta Hospital, Edmonton, Canada; 2 Department of Medical Microbiology and Immunology, University of Alberta, Edmonton, Canada; 3 Department of Laboratory Medicine, National University Hospital, Singapore, Singapore; 4 Department of Mechanical Engineering, The University of Hong Kong, Hong Kong SAR, China; 5 Turning Point Alcohol and Drug Centre, Eastern Health, Melbourne, Australia; 6 Eastern Health Clinical School, Monash University, Melbourne, Australia; 7 School of Public Health, The University of Hong Kong, Hong Kong SAR, China; 8 Saw Swee Hock School of Public Health, National University of Singapore, Singapore, Singapore; 9 Department of Mechanical Engineering, University of Maryland, Baltimore, Maryland, United States of America; 10 Department of Building, School of Design and Environment, National University of Singapore, Singapore, Singapore; 11 Department of Pathology, Yong Loo Lin School of Medicine, National University of Singapore, Singapore, Singapore; 12 Department of Family Medicine and Primary Healthcare, Hong Kong West Cluster, Hospital Authority, Hong Kong SAR, China; 13 University Health Service, The University of Hong Kong, Hong Kong SAR, China; Mount Sinai School of Medicine, United States of America

## Abstract

Two independent studies by two separate research teams (from Hong Kong and Singapore) failed to detect any influenza RNA landing on, or inhaled by, a life-like, human manikin target, after exposure to naturally influenza-infected volunteers. For the Hong Kong experiments, 9 influenza-infected volunteers were recruited to breathe, talk/count and cough, from 0.1 m and 0.5 m distance, onto a mouth-breathing manikin. Aerosolised droplets exhaled from the volunteers and entering the manikin’s mouth were collected with PTFE filters and an aerosol sampler, in separate experiments. Virus detection was performed using an in-house influenza RNA reverse-transcription polymerase chain reaction (RT-PCR) assay. No influenza RNA was detected from any of the PTFE filters or air samples. For the Singapore experiments, 6 influenza-infected volunteers were asked to breathe (nasal/mouth breathing), talk (counting in English/second language), cough (from 1 m/0.1 m away) and laugh, onto a thermal, breathing manikin. The manikin’s face was swabbed at specific points (around both eyes, the nostrils and the mouth) before and after exposure to each of these respiratory activities, and was cleaned between each activity with medical grade alcohol swabs. Shadowgraph imaging was used to record the generation of these respiratory aerosols from the infected volunteers and their impact onto the target manikin. No influenza RNA was detected from any of these swabs with either team’s in-house diagnostic influenza assays. All the influenza-infected volunteers had diagnostic swabs taken at recruitment that confirmed influenza (A/H1, A/H3 or B) infection with high viral loads, ranging from 10^5^-10^8^ copies/mL (Hong Kong volunteers/assay) and 10^4^–10^7^ copies/mL influenza viral RNA (Singapore volunteers/assay). These findings suggest that influenza RNA may not be readily transmitted from naturally-infected human source to susceptible recipients via these natural respiratory activities, within these exposure time-frames. Various reasons are discussed in an attempt to explain these findings.

## Introduction

In recent years, discussions over the most clinically significant routes of influenza transmission have been extensive [1], [2]. Confusion and disagreements surround the definitions of the various transmission routes including ‘close contact’ transmission, ‘airborne’ transmission and ‘droplet’ transmission [3]–[6].

Traditionally in outbreak investigations, airborne transmission has been implicated in secondary cases where direct contact with the infected source has not been documented. Close contact transmission has been used to explain secondary cases arising from documented close contact with the presumed index case [6]. However, it is important to note that even in close proximity, multiple transmission routes may all be responsible for disseminating the infection, i.e. person-to-person transmission in such situations can be potentially due to either airborne, droplet and/or direct physical contact transmission [7], [8].

In the close contact exposure scenario, small droplets generated by an infectious patient can be directly inhaled and deposit in both the upper and the lower airway, whereas large droplets can be also be directly inhaled, but the majority of these will probably deposit in the upper airways only, or directly enter the recipient’s eyes or even the mouth as a direct droplet infection (as opposed to self-inoculated infection) [1], [9]–[12]. Long distance airborne transmission has been postulated to be introduced by small droplet nuclei being carried by ambient airflows, where the moisture from small droplets has mostly evaporated away [7].

The actual clinical and public health implications of these different routes of transmission in everyday situations remains controversial [1], [2], [6], [7], [9], [13], [14], and many researchers have been focusing on the potential for human influenza transmission during real-life activities such as breathing, talking, coughing and sneezing [15]–[18]. These studies have focused on characterising the number, size and content of droplets generated by such activities. Yet this data by itself is not sufficient to determine true transmissibility potential of any viruses carried in these droplets – they still need to reach a susceptible recipient and be inhaled in a sufficient infectious dose (for that individual) to cause infection and disease.

In this paper, two independent studies conducted during 2010, 2011 and 2012 by two different teams are reported, one from Hong Kong and one from Singapore. The two studies had an identical aim, to test the potential for the transmission of influenza from a naturally influenza-infected human to a life-like human manikin ‘recipient’ through real-life respiratory activities, such as breathing, talking, coughing sources. The outcomes of these two studies are presented together due to the similar and largely unexpected results, which showed little or no evidence of detectable influenza (RNA) transmission from the human source to the manikin recipient, with any mode of respiratory activity.

Note that the approach used in these experiments is different from several previous studies in that there is no attempt to capture all exhaled particles – the main focus of these studies is to examine how many of these potentially viral-laden particles actually reach the target manikins.

The Hong Kong study used a shop display manikin, customised for ‘mouth-inhaling’, to examine the quantity of influenza virus inhaled when exposed to a naturally influenza-infected human volunteer source. This study only examined the inhalation phase of a potential recipient. The Singapore study used a commercial thermal, breathing manikin with a full breathing cycle to quantify the amount of influenza virus landing on facial skin sites.

## Materials and Methods

This study was approved by the Institutional Review Board of the University of Hong Kong. Signed informed consent was obtained from all participants. This ethics approval included the participation of children in this study, for whom verbal and written consent was also obtained from their parents, caregivers or guardians, as appropriate. Ethics approval for the Singapore study was granted by the Domain Specific Review Board (DSRB) of the National Healthcare Group (2009/00341) Singapore, and informed verbal and written consent was obtained from each participant in the study.

### Hong Kong experiments (2010–2011)

**Figure 1:**
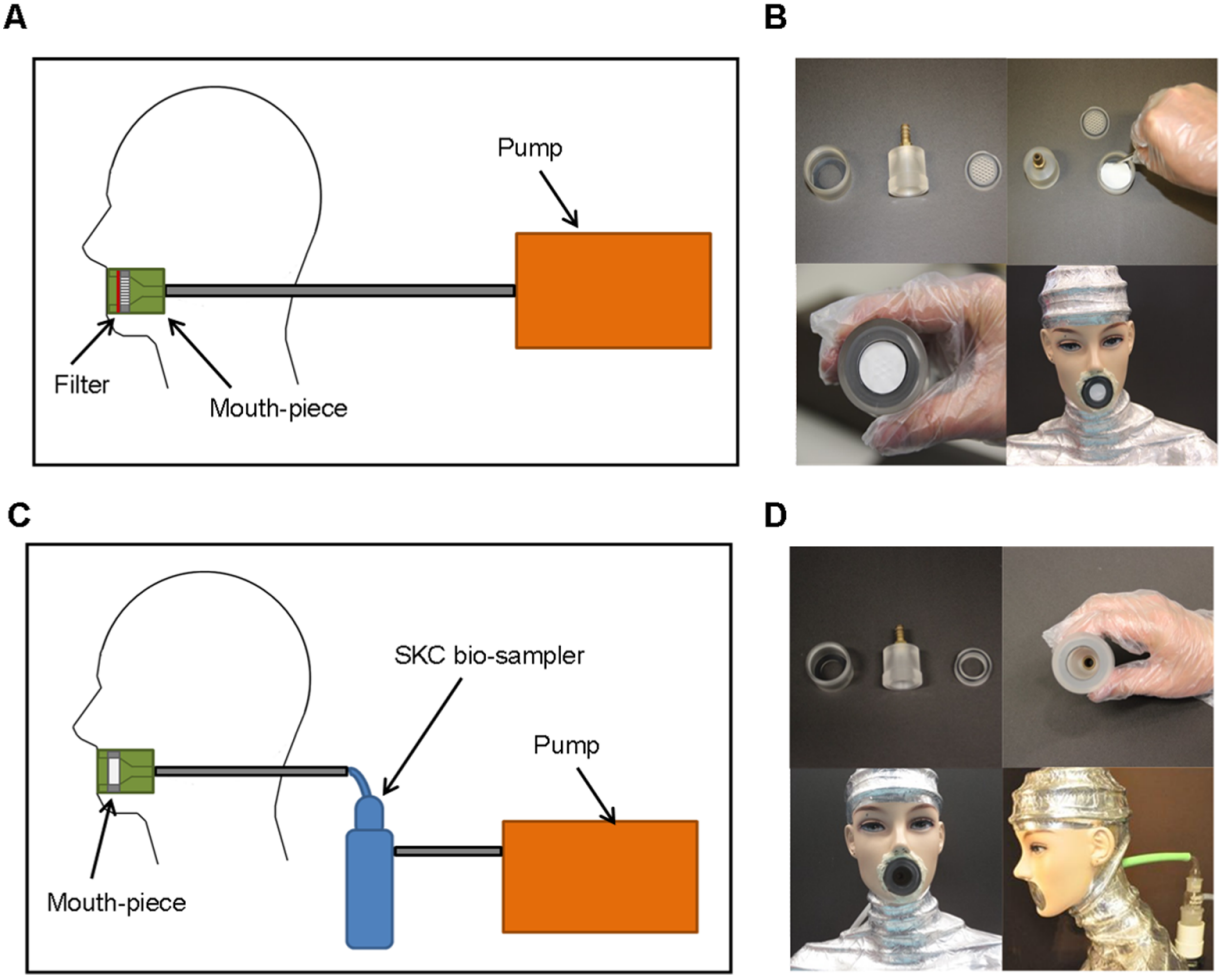
Airborne sampling experimental set-up (Hong Kong experiments), showing: A and B. Design of the mouthpiece with PTFE filter (‘filter’) in place. **C and D**. Installation of the SKC BioSampler, with the mouthpiece, in the manikin.

For these experiments (performed during the 2010–2011 influenza season in Hong Kong SAR), a customised manikin was used as the model of a human recipient. A normal shop display manikin was obtained and a mouth orifice hollowed out, into which was fitted a mouth-piece connected to a pump through the back of the manikin’s neck (**Figure 1**). This pump maintained a continuous inhalation flow of 12.5 L/min, to simulate the inhalation phase of human respiration. The inhalation rate of the manikin was set to twice that of natural human inhalation to create a similar inhalation flow field when both inhalation and exhalation are present. This setting arises from the following argument: if a typical human breath cycle consists of a 50∶50 inhalation:exhalation ratio then the inhalation flow rate is equal to two times the tidal volume times the breathing frequency. The factor of two arises because you are only inhaling half the time, i.e. you multiply the minute ventilation by a factor of two to get the inhalation flow rate because you only spend half the breath cycle inhaling (you are exhaling for the other half) [19], [20]. This also maximized SKC bio-sampler air sampling efficiency.

**Figure 2:**
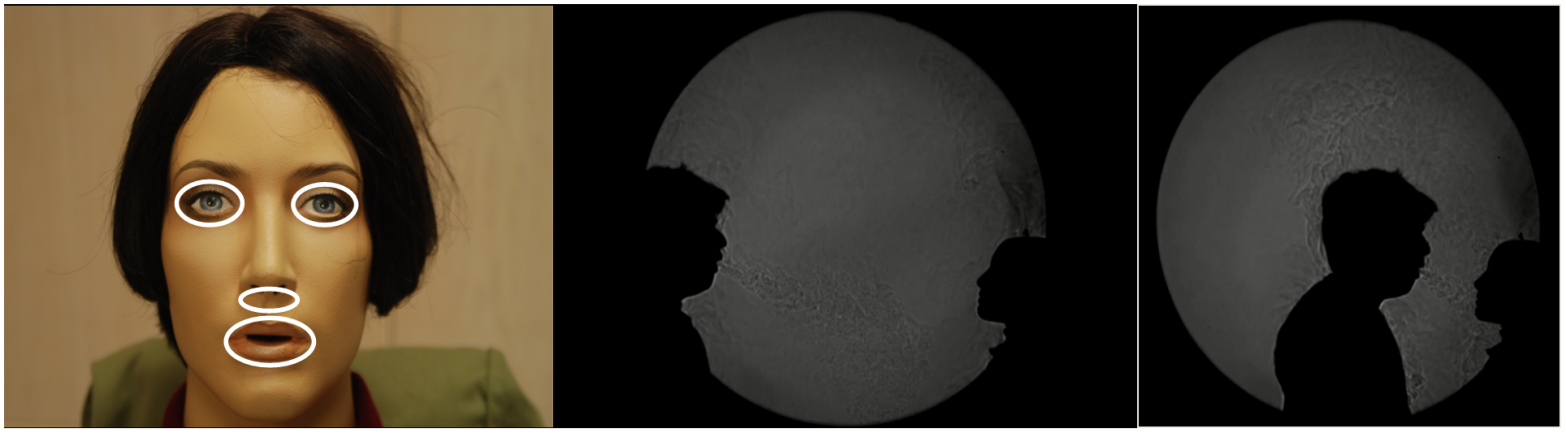
Singapore experimental set-up, showing: Swabbing sites for the manikins’ face (A) for influenza testing. Shadowgraph images of far- (B) and near- cough (C) distances (see accompanying online Video S1 for further details of these shadowgraph images).

Once the manikin and the pump were set up, two methods were used to capture aerosolised virus produced by the naturally influenza-infected volunteers: 1) a polytetrafluoroethylene (PTFE) filter inserted into the mouth orifice of the manikin, to trap any aerosolised virus (**Figure 1A**); 2) and a commercially available air sampler (the 5 ml SKC BioSampler, SKC Inc., Eighty Four, PA USA: http://www.skcinc.com/prod/225-9594.asp) with 2 ml viral transport medium, which was attached to the mouth orifice through the back of the manikin’s neck (Figures 1B and 1C). This air sampler was selected as it has been shown to perform well in collecting influenza-laden air samples effectively [21].

Each of these methods was used separately with each volunteer to capture aerosolised virus. The two methods were not used in combination with any of the volunteers. The reason for the two sampling methods was to allow the capture of large droplets that travelled ballistically as expelled from the source volunteer, transported by the source exhalation airflow (PTFE filters), as well as any smaller, droplet nuclei that were truly airborne, using the SKC BioSampler.

All the PTFE filters were kept in sealed plastic bags to prevent contamination and installed immediately prior to the experiments. Similarly, the mouth-pieces were sterilised and kept in sealed packets and only installed immediately before each experiment with the human volunteers. To capture the virus using the SKC BioSampler, a tissue culture medium (Medium 199, Life Technologies, Kowloon, Hong Kong SAR) was tested and found to be appropriate for the viral capture media. In addition, a baseline efficiency experiment was also performed to ensure that there was minimal loss of detection sensitivity using the PCR method when detecting for the presence of any viral RNA on the PTFE filter.

This study was conducted in a public hospital and a university health clinic during two distinct periods (August to September 2010 and January to February 2011). Patients over 21 years of age with influenza-like illness (ILI: any of cough, fever ≥38°C, sore throat, headache, malaise, myalgia, lethargy) in the previous three days were invited to participate in the study.

A rapid point-of-care test (QuickVue Influenza A+B rapid diagnostic test, Quidel Corp., San Diego, CA, USA: sensitivity: 0.68, specificity: 0.96) [22] was used as instructed by the manufacturer as a screening test, to confirm influenza infection. Patients with positive diagnostic results were then invited for the exhaled breath sampling experiment. Nasal and throat swabs were collected into universal transport medium (UTM, Copan Diagnostics, Murietta, CA, USA) for diagnostic testing using an in-house influenza reverse transcription quantitative polymerase chain reaction (RT-qPCR) assay [23], to further confirm influenza infection and establish a baseline viral load.

Once the manikin was set up, for each sampling method, the recruited, naturally influenza-infected volunteers were asked to perform various respiratory activities (including breathing, counting, talking and coughing) when facing the customised manikin at a distance of 0.1 m and 0.5 m. The overall exposure period of the manikin target to each influenza-infected human volunteer was around 10–15 minutes. After each set of respiratory activities was conducted with the PTFE filter or the SKC BioSampler, the filter or capture media was removed and stored. The PTFE filter was first dipped in 2 mL UTM (universal viral transport medium) prior to storage. All specimens were stored at 2 to 8°C for less than 24 hours before PCR testing to determine the presence and quantity of influenza virus present.

For the PTFE filter samples, briefly, the mouthpiece filter holder was removed carefully and the filter removed. The filter was then soaked in 2 mL of UTM for 15 minutes, during which there were three episodes of vortexing for 30 seconds to transfer as much virus from the PTFE filter to the UTM for influenza RT-PCR testing. From this UTM, 140 µL was used for the RNA extraction step, without further concentration steps. This RT-PCR assay used consensus primers to target the matrix (MP) gene of the virus [23]. Calibrators were included in each run to allow a standard curve to be plotted to estimate the copy numbers in the samples. This assay had a detection limit of approximately 18,000 viral RNA copies/ml UTM.

To check the sensitivity of the PTFE capture method, samples of the PTFE filter were inoculated (by droplets of 1, 5, 25 µL volume) with different, known amounts of influenza RNA, then run through the whole extraction and RT-PCR process for influenza RNA detection. Overall, there was relatively little loss of sensitivity with the log_10_(inoculated) vs. log_10_(detected) viral loads being mostly within 10% of each other.

### Singapore experiments (2011–2012)

For the Singapore experiments, otherwise healthy volunteers with an ILI were recruited from the local university student health centre clinic during the ‘autumn/winter’ period (September 2011 to February 2012). Successful recruits were taken directly to the experimental chamber, where they would expose a life-size, breathing, thermal manikin to various exhaled respiratory airflows. Clinical inclusion criteria included ILI with a temperature of at least 38°C. If the recruits were unable to go straight to the experimental chamber immediately after their clinic visit, they were excluded from the study. Before each experiment, a baseline nasopharyngeal swab (NPS) was taken from each participant for diagnostic testing.

A full-size, commercial thermal, breathing manikin (PT Teknik, Espergærde, Denmark: http://pt-teknik.dk/history) was used as the target for these exposure experiments. Both the manikin’s thermal and breathing modes were turned on, during these exposure experiments, though only the manikin’s face was to be tested for the presence of influenza RNA, after exposure to the naturally influenza-infected volunteers.

Before each exposure session, the manikin’s face was cleaned with medical grade alcohol swabs and allowed to dry for 2–3 minutes. Baseline ‘clean’ swabs were taken from around the manikin’s mouth (1 swab), nose (1 swab) and eyes (1 swab), before, then again after the manikin was exposed to the volunteer performing (Figure 2). The participant performed the following respiratory activities directly (within a 1 m distance) into the face of the manikin: nasal breathing (for 20 seconds), mouth breathing (20 s), counting slowly from one to ten in English (43 s), counting slowly from one to ten in a second language (e.g. Mandarin, German, 43 s), laughing (10 s) and coughing (10 s). Coughing was performed at both far (about ∼1 m) and near (∼0.1 m) distances from the manikin’s face.

The airflow patterns produced during each of these exposure events were visualised and recorded using real-time shadowgraph imaging (**Figure 2** **,** **3** **, Video S1**), using an experimental set-up as previously described elsewhere [24], [25]. In each experiment, for a participant, one diagnostic swab from the participant and 36 facial swabs (representing pre- and post-exposure swabs from each of the eyes, nose and mouth sites), each collected in to 3 mL of UTM, from the manikin were taken for influenza testing. Testing was performed using a routine diagnostic quantitative polymerase chain reaction (qPCR) assay adapted from one already in service [26], [27]. This assay had a detection limit of approximately 3000 viral RNA copies/ml UTM.

**Figure 3:**
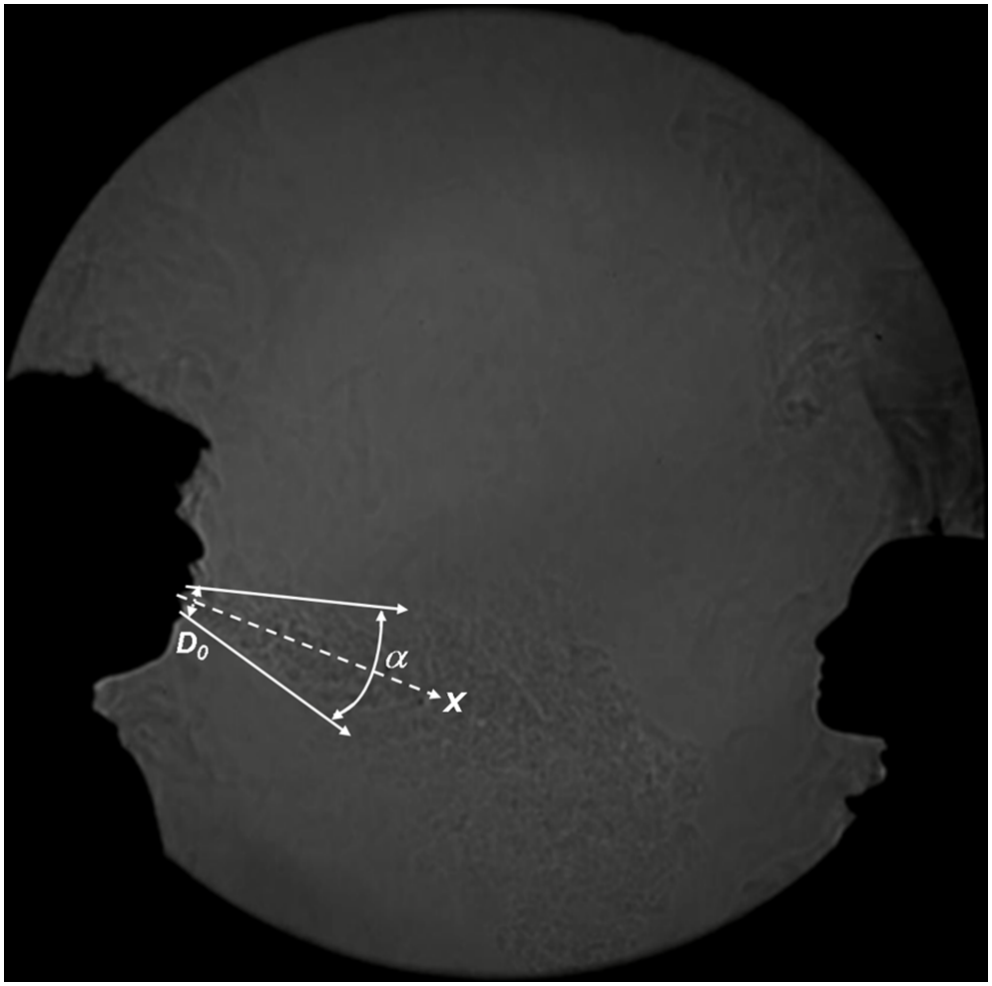
Example of cough shadowgraph image showing the dispersal of the exhaled puff. Parameters that affect the dispersal of this exhaled airflow include the mouth-opening diameter (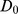), propagation distance (*x*), and spreading angle (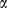) (see accompanying online Video S1 for further details of these shadowgraph images).

## Results

### Hong Kong experiments (2010–2011)

Results were available from 9 volunteers in total, of whom 8 were infected with influenza A and one with influenza B. Each of the volunteers either counted (and/or talked) and/or breathed and/or coughed in various combinations for varying durations. Seven of these volunteers were only 0.1 m from the recipient manikin. The last two volunteers who were exposed to the manikin when only the SKC BioSampler was being used (sampling for airborne droplet nuclei), were also exposed from a larger distance of 0.5 m. Despite the variety of source respiratory activities, the two different sampling methods and exposure distances, no influenza RNA was detected from either the PTFE filter or SKC BioSampler samples from any of the volunteer exposures (**Table 1**).

**Table 1:** Results for the Hong Kong experiments (n = 9).

Subjectcode no.	InfluenzaA/B	Age (yrs)	Sex (M/F)	Days post-onset of illness	Air samplingmethod	Testdistance (m)	Patient ‘source’activities	Influenza RNAdetected infilter/sampler(cop/mL)	Influenza RNAcop/mL in sourcediagnostic swab
00302	A	47	M	3	PTFE filter + SKCBioSampler	0.1	Count 1–20;Cough 10 times	None	9.50×10^7^
01402	A	42	M	3	PTFE filter + SKCBioSampler	0.1	Count 1–100;Cough 10 times	None	1.39×10^5^
01702	A	14	F	2	PTFE filter + SKCBioSampler	0.1	Breath 1 min;Count 1–20;Cough 20 times	None	1.67×10^5^
02602	A	17	F	3	PTFE filter + SKCBioSampler	0.1	Talk 10 min;Count 1–100;Cough 20 times	None	4.19×10^5^
02702	A	22	F	2	PTFE filter + SKCBioSampler	0.1	Talk 10 min;Count 1–100;Cough 20 times	None	8.67×10^6^
03802	A	49	F	3	PTFE filter + SKCBioSampler	0.1	Talk 10 min;Count 1 to 100;Cough 20 times	None	7.40×10^6^
04102	A	57	F	2	PTFE filter + SKCBioSampler	0.1	Talk 10 min;Count 1 to 100;Cough 20 times	None	3.01×10^6^
05602	A	62	F	2	SKC BioSampler	0.1, 0.5	Talk 10 min;Count 1 to 100;Cough 20 times	None	5.38×10^5^
00203	B	not given	M	3	SKC BioSampler	0.1, 0.5	Talk 10 min;Count 1 to 100;Cough 20 times	None	3.70×10^6^

### Singapore experiments (2011–2012)

Out of a total of 23 participants recruited for this study, 6 were diagnosed positive (6/23 = 26%) for influenza virus (2 seasonal A/H3N2, 1 pandemic A/H1N1pdm and 3 influenza B). Most of these volunteers presented within three days of illness onset (3 at 2 days, 1 each at 1, 3 and 6 days post-onset). Despite strongly positive diagnostic PCR results for influenza RNA (range: 4.33–6.83 log_10_) from the nasal swabs of the 6 volunteers, none of the post-exposure swabs taken from the manikin’s face was found to be positive, after exposure to any of the respiratory activities. These manikin swabs were also tested for inhibitory substances to PCR by spiking them with influenza RNA positive control – no PCR inhibition was detected in any of these samples (**Table 2**).

**Table 2:** Results for Singaporean experiments (n = 6).

Subjectcode no.	InfluenzaA/subtype,or B	Age (yrs)	Sex (M/F)	Days post-onset of illness	aTest distance(m) – see footnote	bPatient ‘source’activities – seefootnote	Influenza RNAdetected inmanikin facialswabs (cop/mL)	Influenza RNAcop/mL in sourcediagnostic swab
1	A/H3	22	M	3	1. 1/10	See	None	1.29×10^5^
2	A/H1N1pdm	22	M	2	(o0.1/10.1/1r 0.1for additionalclose-up cough)	footnote*	None	2.88×10^4^
3	B	23	F	6	0.1/1		None	2.14×10^4^
4	A/H3	25	M	2			None	3.55×10^5^
5	B	21	M	1			None	4.57×10^6^
6	B	50	F	3			None	6.76×10^6^

a 0.1 m and 1 m.

b Nasal breathing (for 20 seconds), mouth breathing (20 s), counting slowly from one to ten in English (43 s), counting slowly from one to ten in a second language (e.g. Mandarin, German, 43 s), laughing (10 s) and coughing (10 s). Coughing was performed at both far (about ∼1 m) and near (∼0.1 m) distances from the manikin’s face.

## Discussion

These experiments were conducted to further the understanding of how influenza is transmitted amongst humans. Multiple studies have been published on influenza air-sampling from the environment or human or simulated sources [15]–[18], [28]–[30], as well as influenza transmission between various animal models [31], [32]. Yet there have been few, if any studies focusing on the recipient end of the influenza transmission pathway. One such study by Lindsley et al [30] investigated the effect of wearing a face shield on the viral load potentially inhaled by the wearer, using a simulated coughing patient source and breathing healthcare worker recipient (wearing the face shield). They found that the face shield was highly effective in reducing the amount of virus potentially reaching the recipient by over 90% at separation distances of either 46 cm or 183 cm. However, continued presence in the same chamber would eventually result in a reduction of only ∼80% as dispersion of the smaller, airborne particles in the room would eventually travel around the face mask to be inhaled. It was unclear what the starting aerosolised viral load was in this study.

This investigational stage of the transmission pathway might be termed: ‘end-point host-exposure and sampling’, i.e. what might be *actually* inhaled at the face vs. what might be *potentially* inhaled, based on the larger, air-sampled ‘source’ environment. The lack of any detectable influenza RNA from the swabs taken from the manikin’s face (Singapore experiments) and inhaled breath (Hong Kong experiments) after exposure to infected volunteers, was initially surprising, but became more understandable in light of the study published by Milton and colleagues [18]. Again, note that the approach used in these experiments is different from several previous studies as there is no attempt to capture all exhaled particles. The main aim of these ‘end-point sampling’ studies is to investigate how many of these potentially viral-laden particles actually reach the target manikins.

### Large droplets may be less likely to transmit influenza

In the Singapore experiments, the cycle threshold (Ct) values for the PCR positive NPS samples from the influenza positive recruits were all reasonably low (indicating the presence of a relatively high viral load) in these samples. The real-time shadowgraph video footage taken during these experiments clearly show the cough puff impinging directly onto the manikin’s face in the vicinity of these swabbing sites (**Figure 2** **, Video S1**). Possible explanations for this might be that despite the relatively high influenza load on the NPS samples from these naturally infected recruits, the droplets expelled during these respiratory activities did not carry high numbers of viruses to transmit to the manikin’s face.

More intriguingly is the possibility that the viral-laden saliva/mucous in the oral cavity is not of uniform viscosity, with the more localised immune responses in parts of the mouth (and/or oro-/naso-pharynx) increasing the local viscosity, thus allowing the lower viral load saliva/mucous of lower viscosity being preferentially expelled during respiratory activities. A review by Fabian et al. [33] suggests that salivary mucins, particularly MU7, have a high affinity for and may trap and agglutinate micro-organisms such as bacteria, fungi and viruses. Also another salivary mucin, MUC5b, has been shown to have antiviral properties, and can form hydrophilic viscoelastic gels that can increase the viscosity of saliva.

In the Hong Kong experiments, the results suggest that influenza virus cannot be detected in the inhaled breath after a source exposure from a minimum distance of 10 cm or greater, for these 15 patients. Similarly with the Singapore experiment, it is possible that the influenza virus levels in the exhalation airflows were just too low to be transmitted in detectable quantities to the recipient manikin. In these Hong Kong experiments, after the participant finished talking or coughing, large droplets were normally visible on the filters (diameters around 1–3 mm). These droplets had not evaporated by the time these filters were immersed in the transport media. Yet, influenza virus RNA was still not detectable even in these samples. The detection of little or no influenza RNA in these experiments was initially surprising, but again, maybe compatible with the results of Milton and colleagues [18], who showed maximum copy numbers of <1000 by day 3 of illness in both coarse (>5 µm) and fine (≤5 µm) aerosol particles.

Duguid [34] suggested that large droplets are mostly generated from the anterior mouth. Influenza viruses, however, are rarely found in human saliva (Cowling et al. unpublished data) due to the antiviral substances existing in saliva [35]. Hence there is a possibility that large droplet transmission of influenza may not be important. This may also be true from a different angle. Breathing tends not to produce large droplets [28] and breathing is the most common respiratory activity in humans, so this is probably the most important human respiratory modality that would transmit influenza. Although coughing and sneezing do produce larger droplets, very little time is actually spent coughing and sneezing by most people (though admittedly the frequency of coughing and sneezing may increase with some respiratory infections), so these modalities in general, may not be most important for influenza transmission.

### Dispersion of exhaled aerosols with distance may reduce the likelihood of transmission

These negative results may be also due to low virus concentration at a distance from the source caused by a dispersion effect. This is perhaps one of the more important differences between our experiment and other studies with successful virus recovery [15]–[18], [28]–[29], i.e. that we did not capture the whole exhaled breath volume (regardless of modality, i.e. breathing, talking, coughing, etc.) from the sources. Hence, the total amount of viral RNA that was potentially detectable in these Hong Kong and Singapore studies may not be comparable to these other studies, and will likely be considerably less (see the estimated detectable viral loads in **Video S1**).

This explanation would also apply to the Singaporean experiments, and a qualitative visual confirmation of this dispersal effect can be seen from the shadowgraph images (**Figure 3** **, Video S1**). This figure also suggests the various parameters that are likely to affect the extent of the dispersal (and therefore dilution) of this exhaled airflow, including the mouth-opening diameter (*D_0_*), mean dispersal angle (*α*) and propagation distance (*x*). Previous studies have measured these parameters in human volunteers, giving ranges for the mouth-opening diameter during coughing as *D_0_* = 2.34 cm [36], and mouth-breathing as *D_0_* = 1.23 cm [37], with a mean dispersal angle for mouth-breathing and coughing of *α* = 25–35° (mean 30°) [36], [37]. An exact equation taking into account these parameters, together with the various air mass exchanges across the boundaries of the spreading cone due to turbulent flows, as well as the behaviour of the smaller scale airflows within the spreading cone, is beyond the scope of this article. However, it is clear from **Figure 3** (and **Video S1**) that the final numbers of droplets (and any virus that they might be carrying) arriving at the recipient’s inhalation zone are likely to be considerably lower than that which left the source.

Another point to note from the shadowgraph images shown in **Figures 2** **and** **3** **(and Video S1)** is that although the volunteers were asked to breathe, talk, cough and laugh directly towards the manikin from various distances, their natural, involuntary head movements (particularly during coughing and laughing) were not controlled in any way. These head movements, together with the dispersion factor described above, may have also acted to reduce the amount of virus landing on the manikin’s face in both studies. However, these head movements were deliberately kept as natural as possible to present realistic exposure scenarios for these experiments.

Despite the sensitivity of RNA detection by the PCR method, this dispersal and accompanying dilution (with ambient air) effect may combine to make it difficult to detect any influenza RNA at the manikin - either directly landing on the facial surface (as shown in the Singapore experiments), or within the inhaled airborne particles captured by the filter or the air sampler (as shown in the Hong Kong experiments).

### Totality of particle/droplet capture and durations of exposure

Another possible reason for the lack of detection of any influenza RNA is the relatively short duration of exposure: about 15 minutes in the Hong Kong and 10 seconds to a few minutes in the Singapore experiments. In addition, for the Singapore experiments, only a small rim was swabbed around the eyes, under the nose and around the mouth of the manikin, which may have reduced the amount of detectable virus in these experiments. However, to some extent, it was one of the aims of these experiments to assess what the likely influenza transmission risk would have been, given naturally occurring respiratory activities in everyday situations. Other studies have detected low levels of virus in exhaled particles from coughing and breathing in enclosed chambers for complete particle exposure/inhalation counts [15], [17], [28], [29], with longer exposure and collection times of up to 20–30 minutes [15], [29], but these are artificial experimental situations. In naturally occurring exposure scenarios, dispersion and dilution of these exhaled particles is quite normal and these experiments were designed to test how much viral RNA was detectable at the manikin’s face, in spite of these dispersal and dilution factors. Two studies that investigated coughing into a closed chamber found that very little virus can be found in droplets produced by coughing (<50 viral RNA copies per cough [17], despite significant numbers of droplets being produced during coughing whilst infected with influenza (mean number 75,400/cough, median 46,400/cough, s.d. 97,300/cough, [28]). However, the diagnostic influenza viral load by qPCR from nasopharyngeal swabs collected from the infected volunteers in the former study [17] was reported as being very low (median viral copy number of 51 per sample). In this study, the diagnostic ‘source’ viral RNA copy numbers were much higher than this, so it might be expected that the amount of virus reaching the target manikins would be much higher.

### Possible limitations of the sampling and detection methods

In the Singapore experiments, an alternative possible explanation might be that the surface of the manikin’s face may have been too smooth to capture these airborne droplets (unlike human facial skin and mucous membranes) and that even if the droplets were carrying significant numbers of viruses, the droplets simply ‘bounced’ off the manikin’s face, without leaving any detectable influenza RNA. From the shadowgraph imaging, it is clear that exhalation flows certainly impact upon the manikin’s face (this is especially obvious with the close-up coughing from about 0.1 m distance). For these Singapore experiments that used a thermal manikin (with a surface skin temperature similar to that of a human – around 33–35°C), the generated thermal plume may be an additional factor that could have reduce the amount of airborne virus actually settling on the manikin’s skin surface. The human thermal plume has been described in various studies, and may act as a sort of natural, protective air curtain in this regard [38]–[40]. Perhaps both of these reasons may be relevant in these experiments, and further studies are required to resolve this issue.

In the Hong Kong experiments, a pump was used to extract air through filters, which will increase the evaporation rate of droplets deposited on the filter. Together with the accompanying shear stresses applied to the influenza virus (which is a relatively labile, lipid-enveloped RNA virus), this would also decrease the virus survival rate on the filter, though this should not significantly affect the PCR detection sensitivity of viral RNA. However, the SKC BioSampler also has limitations in that its collection efficiency decreases significantly with increasing particle diameter from about 100% with 4 µm particles to about 30% with 9µm particles [41]. This may underestimate the viral loads detected in larger particles. With the PTFE filter capture and detection methods, any loss of sensitivity was relatively limited, with the log_10_(inoculated) vs log_10_(detected) viral loads being mostly within 10% of each other, according to the baseline experiments.

### Comparison with other similar studies

Two other studies by Bischoff and colleagues have estimated approximate viral concentrations at certain distances. A study on the potential transocular transmission of influenza suggested that exposure to aerosolised influenza at a distance of up to 1 foot, would be sufficient to inoculate (i.e. infect) most exposed human subjects via the ocular route [42]. However, this exposure may bear little resemblance to a natural exposure with wild-type seasonal influenza virus, as it consisted of a 20-minute exposure to a mechanical aerosol generator emitting a mono-dispersed aerosol (of approximately 4.9 mm diameter) of the live attenuated vaccine (‘Flumist’) strain of influenza. The virus concentrations of this artificially generated aerosol and the naturally generated aerosols are difficult to compare.

Bischoff et al. [43] subsequently attempted to define concentration contours around patients infected with influenza, using Andersen samplers to sample air at head level distances of ≤0.305 m (1 ft), 0.914 m (3 ft) and 1.829 m (6 ft) away the heads of influenza infected patients. The upper limits of the viral concentration measured at 0.305 m and 0.914 m were roughly similar at approximately 400–600 and 350–600 “influenza virus RNA copies per 10-L human respiratory minute volume”. Although this unit is difficult to compare to the results of the Hong Kong and Singapore experiments exactly, it does seem to agree with the implications of Milton et al. [18] that the airborne viral load exhaled by infected patients/volunteers is not particularly high, and together with the dispersion calculations above, may well result in very little virus actually reaching and depositing within the breathing zone of a susceptible recipient up to 1 m away.

In summary, these two complementary sets of experiments from Hong Kong and Singapore, with naturally-infected human volunteers, exhaling in various respiratory modalities, directly onto manikin targets, resulted in no detectable transmission of influenza virus RNA. Taking into account other recent findings of the relatively low viral loads in aerosolised droplets [18], this suggests that influenza may not be particularly transmissible by the aerosol route in most circumstances. Further experiments are required to confirm these findings. However, this does not exclude the possible transmission of the virus in situations with longer exposure/contact periods, or in super-spreader individuals who may well shed higher levels of virus in aerosolised form.

## Supporting Information

Video S1 **A 21-year old male coughing onto the target thermal, breathing manikin from 0.1 m then 1 m distance.** Images shot at 2000 frames-per-second (fps) on a Photron SA1.1 high-speed camera. Playback speed: 100 fps (about one quarter normal speed) for clarity.
(WMV)
